# Description of New and Amended Clades of the Genus *Photobacterium*

**DOI:** 10.3390/microorganisms6010024

**Published:** 2018-03-12

**Authors:** Alejandro M. Labella, M. Dolores Castro, Manuel Manchado, Juan J. Borrego

**Affiliations:** 1 Department of Microbiology, Faculty of Sciences, Universidad de Malaga, 29071 Malaga, Spain; amlabella@uma.es (A.M.L.); dcastro@uma.es (M.D.C.); 2 Puerto de Santa María, Junta de Andalucía, IFAPA Centro El Toruño, 11500 Cadiz, Spain; manuel.manchado@juntadeandalucia.es

**Keywords:** *Photobacterium* species, clades, MLSA, phylogenetic study, 16S rRNA

## Abstract

Phylogenetic relationships between species in the genus *Photobacterium* have been poorly studied despite pathogenic and ecological relevance of some of its members. This is the first phylogenetic study that includes new species of *Photobacterium* (validated or not) that have not been included in any of the previously described clades, using 16S rRNA sequences and multilocus sequence analysis (MLSA) in concatenated sequences of *gyrB*, *gapA*, *topA*, *ftsZ* and *mreB* housekeeping genes. Sequence analysis has been implemented using Maximum-parsimony (MP), Neighbour-joining (NJ) and Maximum likelihood (ML) treeing methods and the predicted evolutionary relationship between the *Photobacterium* clades was established on the basis of bootstrap values of >75% for 16S rRNA sequences and MLSA. We have grouped 22 species of the genus *Photobacterium* into the following 5 clades: Phosphoreum (comprises *P. aquimaris*, “*P. carnosum*,” *P. iliopiscarium*, *P. kishitanii*, *P. phosphoreum*, “*P. piscicola*” and “*P. toruni*”); clade Profundum (composed of *P. aestuarii*, *P. alginatilyticum*, *P. frigidiphilum*, *P. indicum*, *P. jeanii*, *P. lipolyticum*, “*P. marinum*,” and *P. profundum*); clade Damselae (two subspecies of *P. damselae*, *damselae* and *piscicida*); and two new clades: clade Ganghwense (includes *P. aphoticum*, *P. aquae*, *P. galatheae*, *P. ganghwense*, *P. halotolerans*, *P. panuliri* and *P. proteolyticum*); and clade Leiognathi (composed by *P. angustum*, *P. leiognathi* subsp. *leiognathi* and “*P. leiognathi* subsp. *mandapamensis*”). Two additional clades, Rosenbergii and Swingsii, were formed using a phylogenetic method based on 16S rRNA gene, although they are not confirmed by any MLSA methods. Only *P. aplysiae* could not be included in none of the established clade, constituting an orphan clade.

## 1. Introduction

The family *Vibrionaceae* (Gammaproteobacteria) is a diverse group of Gram-negative bacteria that includes following genera: *Vibrio*, *Photobacterium*, *Aliivibrio*, *Catenococcus*, *Echinomonas*, *Enterovibrio*, *Grimontia* and *Salinivibrio* [1,2]. According to divergence in the 16S rRNA gene sequence and phenotypic characteristics, the genus *Photobacterium* nowadays comprises more than 28 validated species [3], which are widespread both in the marine environment (seawater, sediments and marine animals) and in saline lakes [4]. Some species are bioluminescent and form specific bioluminescent mutualisms with marine fish [5]. In addition, several strains have been reported to be pathogenic for both poikilothermic and homeothermic animals and are capable of producing important disease outbreaks with a high economic impact [4,6]. 

*Photobacterium* species display varied phenotypic, physiological and ecological characteristics, although all of them are chemoorganotrophs, possess Q-8 as the predominant respiratory lipoquinone and present C_16:1_ and C_16:0_ as their major fatty acids. Recently, this genus has been revised on the basis of its taxonomic, ecological and pathogenic characteristics [4]. The type species of the genus, *Photobacterium phosphoreum*, was included in the Approved Lists of Bacterial Names [7] together with *P. angustum*, *P.* (*Aliivibrio*) *fischeri* and *P. leiognathi*. *P. damselae* [8] was formed as a new combination for former *Vibrio damsela* and *Pasteurella piscicida*. This species is the only one for which subspecies have been proposed so far with the publication of *P. damselae* subsp. *piscicida* [9]. Moreover, *P. damselae* subsp. *damselae* is an earlier heterotypic synonym of *P. histaminum* [10]. In the last two decades, the number of descriptions has intensified with the proposal of 24 novel species and two new combinations with valid names (Table 1). 

Multilocus sequence analysis (MLSA) of genes coding for housekeeping proteins is particularly useful for epidemiological studies, for resolving taxonomic ambiguity and for establishing relationships between taxa [11,12]. In addition, MLSA has been used to examine evolutionary relationships within the *Aliivibrio*, *Photobacterium* and *Vibrio* genera and to establish new clades within genus *Aliivibrio* [2,13]. Zeigler [14] pointed out the criteria that genes selected as phylogenetic markers should fulfil: (i) They must be widely distributed among genomes; (ii) they must be present as a single copy within a genome; (iii) the gene sequence must be long enough to contain sufficient information (between 900 and 2250 nucleotides); and (iv) the sequences must predict whole-genome relationships with acceptable precision and accuracy that correlate well with the 16S rRNA data and with whole-genome similarities measured by DNA-DNA hybridization. 

In the case of *Photobacterium* genus, MLSA has been applied in the study of the intragenic relationships of its species into clades using different housekeeping genes. On the basis of 16S rRNA gene sequence, several authors established 3 clades: Clade 1 grouped *P. phosphoreum* and the bioluminescent species *P. angustum*, *P. aquimaris*, *P. iliopiscarium* and *P. kishitanii*; clade 2 was constituted by *P. aplysiae*, *P. frigidiphilum*, *P. indicum*, *P. lipolyticum* and *P. profundum*; and, in clade 3 are included *P. ganghwense*, *P. halotolerans*, *P. lutimaris* and *P. rosenbergii*. The inclusion of *P. leiognathi* and *P. damselae* was uncertain and therefore, they were not included in any clade [15,16,17,18]. Moreover, Urbanczyk et al. [19] using the *lux* operon gene sequences and MLSA analysis, proposed two well-supported clades into genus *Photobacterium*: clade 1 that included the luminous and symbiotic species of *Photobacterium*, such as *P. angustum*, *P. aquimaris*, *P. kishitanii*, *P. leiognathi*, *P. mandapamensis* and *P. phosphoreum* and the non-luminous species, *P. iliospicarium*. Clade 2 grouped most non-luminous species of the genus, such as *P. lipolyticum*, *P. profundum*, *P. frigidiphilum*, *P. indicum*, *P. damselae*, *P. jeanii*, *P. ganghwense*, *P. halotolerans*, *P. gaetbulicola*, *P. lutimaris* and *P. rosenbergii*. The inclusion of *P. aplysiae* in the established clades was uncertain. More recently, Sawabe et al. [20] revised the *Vibrio* clades by MLSA using 9 housekeeping genes (16S rRNA gene, *gapA*, *gyrB*, *ftsZ*, *mreB*, *pyrH*, *recA*, *rpoA* and *topA*) and proposed 8 new clades, four of each included species of genus *Photobacterium*: clade Damselae with two subspecies of *P. damselae*; clade Phosphoreum with four species: *P. angustum*, *P. iliospicarium*, *P. leiognathi* and *P. phosphoreum*; clade Profundum that included the species: *P. lipolyticum*, *P. profundum* and *P. indicum;* and clade Rosenbergii containing only two species: *P. lutimaris* and *P. rosenbergii*. 

However, since 2010, a total of 17 new species belonging to *Photobacterium* genus have been described, such as *P. gaetbulicola* [21], *P. jeanii* [22], *P. aphoticum* [23], *P. atrarenae* [24], *P. swingsii* [25], *P. marinum* [26], *P. aestuarii* [27], *P. aquae* [28], *P. panuliri* [29], *P. piscicola* [30], *P. sanctipauli* [31], *P. galathea* [32], *P. sanguinicancri* [33], *P. proteolyticum* [34], *P. alginatilyticum* [35], *P. toruni* [36] and *P. carnosum* [37]. Until to date, these newly described species are not included in any of the described clades.

Therefore, the aim of this study is to elucidate the phylogenetic relationship of 53 strains belonging to all species reported in genus *Photobacterium*, to validate current reported clades and the clustering of newly described species by using of the 16S rRNA gene sequence and MLSA analysis using 5 housekeeping genes (*gyrB*, *gapA*, *topA*, *ftsZ* and *mreB*). 

## 2. Materials and Methods

### 2.1. Strains and Culture Conditions

A total of 53 *Photobacterium* strains, including type strains and environmental isolates were analysed. Fourteen strains presumptively belonging to the genus *Photobacterium* were isolated from captive fish with signs of disease [38,39]. Strains were routinely cultured on Tryptic soy agar or broth (TSA or TSB) (Difco) supplemented with 1.5% (*w*/*v*) NaCl (TSAs or TSBs, respectively) and incubated at 22 °C for 2 to 5 d. Stock cultures were stored at −80 °C in TSBs with 15% (*v*/*v*) glycerol. 

### 2.2. Phenotypic Characterization

Phenotypic characterization of the 18 *Photobacterium* strains isolated from diseased fish was performed as described previously [40]. Briefly, the following tests were performed: motility and cell morphology, catalase and oxidase activities, oxidation/fermentation test, Voges-Proskauer, utilization of citrate, arginine dihydrolase, lysine- and ornithine decarboxylation, nitrate reduction, indole and H_2_S production, sensitivity to vibriostatic agent pteridine (0/129, 150 μg), and urease, beta-galactosidase, amylase, alginase, gelatinase, lipase and haemolysin production. The strains were grown on TSB to determine salt tolerance between 0.5% and 10% NaCl and grow at different temperatures: 4, 20, 30, 35 and 40 °C. Acids production was investigated on the following substrates: d-mannitol, d-sorbitol, l-rhamnose, sucrose, melibiose, l-arabinose, myo-inositol, d-xylose, d-ribose, d-fructose, d-cellobiose, d-glucose, d-galactose, d-mannose, lactose, maltose, d-trehalose and d-raffinose. Further characterization and confirmation was achieved using API 20E and API 20NE microplates (bioMerieux, Madrid, Spain). The utilization of different substrates as sole carbon and energy sources was determined using Biolog GN2 microplates (Biolog Inc., Hayward, CA, USA). 

### 2.3. DNA Extraction and PCR Amplification 

**Figure 1:**
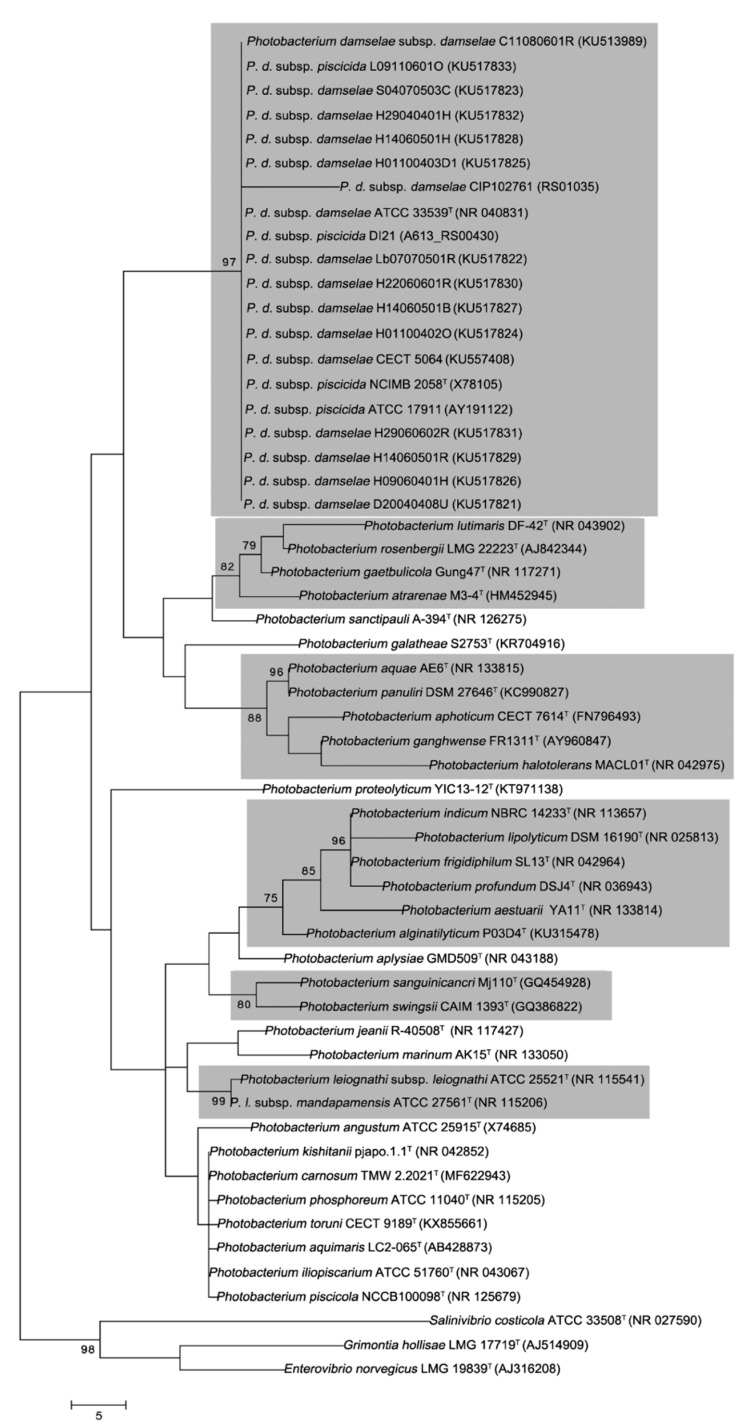
Maximum Parsimony phylogenetic tree based on 16S rRNA gene sequences of environmental and type strains of *Photobacterium* species. *Grimontia hollisae*, *Enterovibrio norvegicus* and *Salinivibrio costicola* type strain sequences have been added as outgroup. Sequence accession numbers are given in parentheses. Bootstrap values greater than 75% confidences are shown at branching points (percentage of 1000 resamplings). Bar indicates number of substitutions per position. Shaded gray delimited the clades defined.

Bacterial genomic DNA was extracted according to methodology described previously [38]. Six genes were studied: *gyrB* (DNA gyrase B subunit), *gapA* (glyceraldehyde-3-phosphate dehydrogenase A), *topA* (DNA topoisomerase I), *ftsZ* (GTP-binding tubulin-like cell division protein), *mreB* (cell wall structural complex MreBCD) and 16S rRNA. PCR primers used for amplification and sequencing of these genes are listed in Supplementary Table S1. PCR amplification was carried out in a 25-μL reaction mixture containing 5 pmol of each primer, 200 μM of each dNTP, 1× PCR buffer (Promega), 2 mM MgCl_2_, 1.5 U Bio*Taq* polymerase (Bioline, London, UK) and 1 μL of bacterial template DNA. PCR amplifications were performed using a Mastercycler thermocycler (Eppendorf, Hamburg, Germany). The conditions for 16S rRNA gene amplification were 1 min at 95 °C, 30 cycles of 1 min at 95 °C, 1 min at 50 °C and 1 min 30 s at 72 °C and a final step of 5 min at 72 °C. For *gapA*, *ftsZ* and *gyrB* gene amplifications, the thermal program consisted of 2 min at 95 °C, 30 cycles of 1 min at 95 °C, 1 min at 50 °C and 10 min at 72 °C and a final step of 5 min at 72 °C. For *mreB* gene amplification, the thermal program consisted of 2 min at 95 °C, 30 cycles of 1 min at 95 °C, 1 min at 55 °C and 5 min at 72 °C and a final step of 5 min at 72 °C. Finally, for *topA* gene amplification, the thermal program consisted of 2 min at 95 °C, 30 cycles of 1 min at 95 °C, 1 min at 60 °C and 5 min at 72 °C and a final step of 5 min at 72 °C. Amplified products were visualized by agarose gel electrophoresis (1.2%) with ethidium bromide staining. Purified PCR amplicons were sequenced using a Bigdye Terminator v.3.1 kit (Applied Biosystems, Foster City, CA, USA) in a 377 DNA sequencer (Applied Biosystems) and Seqman v5.53 (DNASTAR, Thermo Fisher Scientific, Madrid, Spain). GenBank accession numbers of gene sequences used in this study are listed in Figure 1 and Supplementary Table S2.

### 2.4. Phylogenetic Data Analysis 

The sequences of 16S rRNA (*n* = 53) and the *gyrB*, *gapA*, *topA*, *ftsZ*, *mreB* genes (*n* = 47) for MLSA of all strains of *Photobacterium* tested were aligned using MUSCLE software [41]. For MLSA analysis individual genes were aligned and then concatenated using FaBox software [42]. Sequence sizes (nucleotide number) of each of the five housekeeping genes analysed in this study were 2421, 774, 2653, 1143 and 1044 nt for *gyrB*, *gapA*, *topA*, *ftsZ*, *mreB* genes, respectively.

Recombination events in sequence alignments were analysed using the Recombination Detection Program Beta v4.95 (RDP4) [43], following the methods previously described [44,45]. Phylogenetic analysis was performed using the program MEGA6 [46]. Maximum-parsimony (MP) analysis was performed for all gene fragments and the tree was obtained using the Subtree-Pruning-Regrafting (SPR) algorithm [47]. For comparative purpose, phylogenetic analyses using Neighbour-joining (NJ) and Maximum likelihood (ML) treeing methods were carried out using, in both cases, the Jukes-Cantor model [48]. In all cases, gaps and missing data treatment was accomplished using complete deletion strategy. Bootstrap analyses were performed using 1000 replications and a bootstrap of ≥75% was used to provide the confidence estimation for clades in the phylogenetic tree. 

## 3. Results and Discussion

### 3.1. Phenotypic Characterization

Phenotypic characterization of the 18 *Photobacterium* strains isolated from diseased fish is shown in Supplementary Table S3. According to the biochemical and physiological profiles, 15 strains belong to *P. damselae* subsp. *damselae* and 3 strains were classified as *P. damselae* subsp. *piscicida*. The intraspecific variation among two subspecies was obtained for the following features: motility; nitrate reduction; acids from: sucrose, melibiose, d-cellobiose, maltose and d-trehalose; and for the utilization as unique carbon and energy source of the following substrates: glycogen, tween-40, *N*-acetyl-d-galactosamine, β-methyl-d-glucoside, d-raffinose, d-sorbitol, succinic acid, d-l-lactic acid, bromosuccinic acid, succinamic acid, l-alanyl glycine, l-asparagine, l-aspartic acid, l-glutamic acid, l-serine, glycerol and α-d-glucose 1-phosphate. On the other hand, the intraspecific variation among *P. damselae* subsp. *damselae* strains was recorded for the following characteristics: lysine decarboxylase, acetoine production, amylase, gelatinase, lipase and haemolysin production; whilst, for *P. damselae* subsp. *piscicida* strains the intraspecific variation was obtained for acetoine production, amylase and acids from l-arabinose (Supplementary Table S3). The profiles of API 20E for *P. damselae* subsp. *damselae* were 2014144, 2015144, 6014144 and 6015144, while for *P. damselae* subsp. *piscicida* were 2004024, 2004025 and 2005025. In the case of API 20NE, the profiles for *P. damselae* subsp. *damselae* strains were 5342334 and 5342344 and for *P. damselae* subsp. *piscicida* strains the profile was unique 4142344. As it can be seen, the phenotypic patterns are very variable among the strains of the same subspecies and therefore, they are not adequate to apply for evolutionary or phylogenetic studies. 

### 3.2. Phylogenetic Studies of the Photobacterium Genus

**Figure 2:**
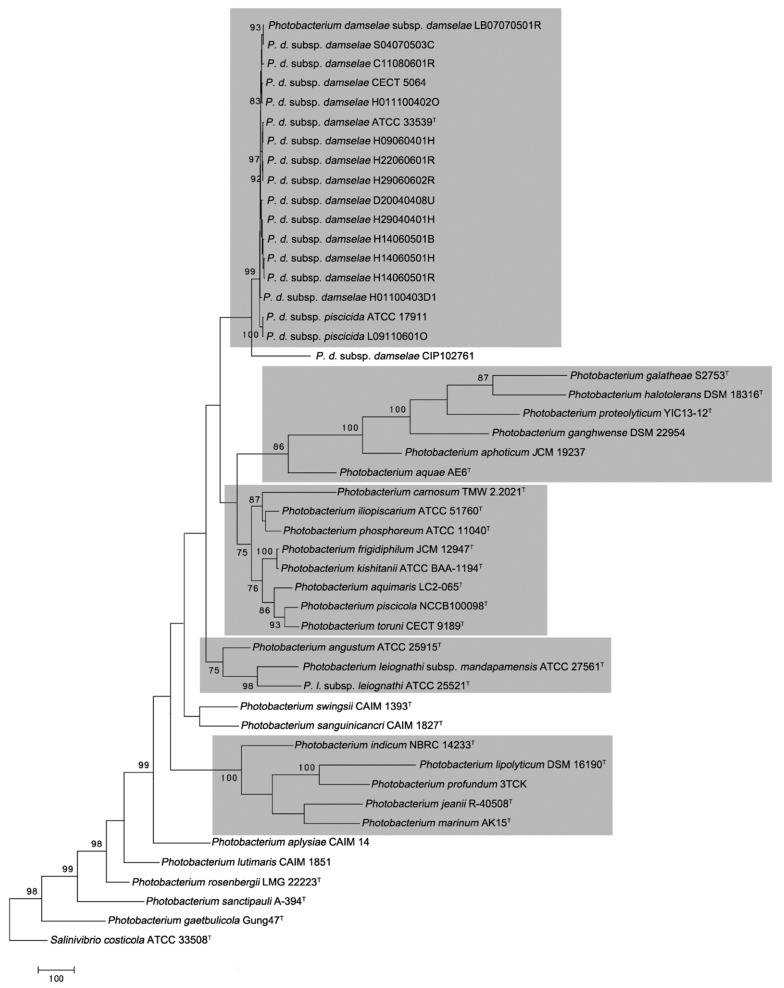
Maximum Parsimony phylogenetic tree based on 5 concatenated genes (*gyrB*, *gapA*, *topA*, *ftsZ*, and *mreB*) of environmental and type strains of *Photobacterium* species. *Salinivibrio costicola* was included as outgroup. The tree is drawn to scale, with branch lengths measured in the number of substitutions per site. Bootstrap values greater than 75 % confidences are shown at branching points (percentage of 1,000 resamplings). Shaded gray delimited the clades defined.

The results of the phylogenetic analysis performed on the 16S rRNA gene sequences are shown in Figure 1 and Supplementary Figures S1 and S2. All strains identified as either *P. damselae* subsp. *damselae* or *P. damselae* subsp. *piscicida* cluster in a single, tightly packed group with 97.0% bootstrap support for MP, with 95.0% for NJ and ML treeing methods (Figure 1, Supplementary Figures S1 and S2, respectively). No internal boundaries appeared between both subspecies, since their sequences display almost no variation and form a tight monophyletic branch constituting a homogeneous group. For a more robust phylogenetic analysis, a MLSA approach using set of five housekeeping genes was performed, which have been proven to be useful for taxonomic and phylogenetic studies of the *Vibrionaceae* family [20,49,50,51,52]. MLSA using MP grouped 17 from 18 strains of *P. damselae* with a bootstrap value of 99% (Figure 2), constituting the clade Damselae. This result is confirmed by the use of NJ and ML treeing methods, including all the 18 strains of *P. damselae*, both with a bootstrap value of 94% (Supplementary Figures S3 and S4). 

The position of other *Photobacterium* species studied on the basis of 16S rRNA and MLSA was always external to the Damselae clade, constituting five additional clades that include all the other 21 *Photobacterium* from 30 described species (Figure 2). Clade Ganghwense, a new proposed clade, includes six species: *P. aphoticum*, *P. aquae*, *P. galatheae*, *P. ganghwense*, *P. halotolerans* and *P. proteolyticum* on the basis of the MLSA approach at a bootstrap value of 86% (Figure 2). In addition, *P. panuliri* may be included in this clade on the 16S rRNA gene sequence at bootstrap values of 88, 86 and 88% for MP, NJ and ML treeing methods (Figure 1, Supplementary Figures S1 and S2). This clade grouped species that possessed <5% GC (mol %) (48.6 ± 3.4%) [15,16,23,28,29]. This result is consistent with that obtained by Lucena et al. [23], who reported that *P. aphoticum* presented a close relationship with *P. halotolerans* and *P. ganghwense* and with the results of Liu et al. [28] who found that *P. aquae* and *P. aphoticum* had higher 96% 16S rRNA sequence similarity. On the other hand, Rivas et al. [16] found that *P. halotolerans* presented a close relationship with *P. ganghwense* and Gomez-Gil et al. [25] established a clade formed by *P. ganghwense*, *P. halotolerans*, *P. aphoticum*, *P. panuliri* and *P. aquae* but *P. galatheae* formed an orphan clade. However, the later species was closely related to *P. halotolerans* according to the results of Machado et al. [32].

Clade Phosphoreum that consisted of eight species: *P. aquimaris*, “*P. carnosum*,” *P. frigidiphilum*, *P. iliopiscarium*, *P. kishitanii*, *P. phosphoreum*, “*P. piscicola*” and “*P. toruni*” (Figure 2). These species possess a DNA-DNA hybridization percentage higher than 20% (from 24.0% to 84.0%) and a GC (mol %) difference lower than 5% of (40.9 ± 1.95%) [12,17,27,31,51,52]. Previously, Park et al. [15] included in the clade Phosphoreum the species *P. phosphoreum*, *P. angustum* and *P. iliopiscarium*, excluding to *P. leiognathi* subsp. *leiognathi* (orphan clade), although the later species was included in this clade by other authors [16,17]. Later, Yoshizawa et al. [18], in the description of *P. aquimaris* performed two phylogenetic analyses based on 16S rRNA and *luxA* genes, revealing the closest phylogenetic relationship with *P. phosphoreum*, *P. iliopiscarium* and *P. kishitanii* and a more distant relationship with *P. angustum* and *P. leiognathi* subsp. *leiognathi*. However, the results obtained in the present study are not coherent with those obtained by Sawabe et al. [20], who using 9 housekeeping genes established the clade Phosphoreum with the species: *P. phosphoreum*, *P. angustum*, *P. iliopiscarium* and *P. leiognathi.*

Clade Leiognathi, is a new proposed clade, consists of two subspecies: *P. leiognathi* subsp. *leiognathi* and *P. leiognathi* subsp. *mandapamensis* and *P. angustum* according to the MLSA at a bootstrap value of 75% (Figure 2). This result is similar to those reported by other authors [5,18].

Clade Profundum comprises five species: *P. indicum*, *P. jeanii*, *P. lipolyticum*, *P. marinum* and *P. profundum* according to the MLSA approach with a bootstrap value of 100% (Figure 2). This clade grouped species other species such as *P. aestuarii*, *P. alginatilyticum* and *P. frigidiphilum* according to the results obtained using the 16S rRNA gene sequence (Figure 1), at a bootstrap value of 75% for any methods used (MP, NJ or ML). The species grouped in this clade shared a percentage DNA-DNA hybridization higher than 12% (from 12.0% to 15.0%) and possessed a difference in the GC (mol %) lower than 5% (43 ± 2.05%) [27,35,53,54,55,56]. This result agrees with that obtained by several authors, using 16S rRNA gene sequence or analysis of concatenated housekeeping genes [52,53,54,57]. Sawabe et al. [20] described the clade Profundum including *P. profundum*, *P. indicum* and *P. lipolyticum* and Urbanczyk et al. [52] included in this group, in addition, the species *P. frigidiphilum* and *P. aplysiae*.

The inclusion of *P. frigidiphilum* into the clade Profundum (16S rRNA gene sequence analysis) or into the clade Phosphoreum (MLSA analysis) is uncertain, although this species has been related to *P. indicum* (clade Profundum) by several authors [15,16,17,52]. The unexpected close proximity of *P. frigidiphilum* to *P. kishitanii* in the concatenated tree (Figure 2), which is due to the higher similarity shared in the housekeeping genes (98.0% *gyrB*, 99.3% *mreB*, 99.5% *topA*, 100% *gapA* and 100% *ftsZ*) than observed for the 16S rRNA gene sequences (97.1%). In future studies with *P. frigidiphilum* should be considered a reassessment of the sequences available.

On the basis of the 16S rRNA gene sequence analysis, four species: “*P. atrarenae*,” *P. gaetbulicola*, *P. lutimaris* and *P. rosenbergii* clustered on the basis of 16S rRNA gene sequences at a bootstrap of 82% (Figure 1) constituting the clade Rosenbergii. However, applying the MLSA approach these species and *P. sanctipauli* formed paraphyletic branches and they could not be included in a clade (Figure 2 and Supplementary Figures S3 and S4), except in the case of the use of NJ methods that grouped *P. lutimaris* and *P. rosenbergii* in a cluster with a bootstrap value of 96% (Supplementary Figure S3), result similar to that reported by other authors [20]. These species shared >20% DNA-DNA hybridization (from 21.5% to 22%) and possessed <5% GC (mol %) (49.7 ± 3.9%) [17,21,24,49]. Results that are partially similar to those reported by several authors [24,52], who reported that the highest degree of similarity of *P. atrarenae* was with *P. rosenbergii* and *P. gaetbulicola*.

The species: *P. sanguinicancri* and *P. swingsii* on the basis of exclusively of 16S rRNA gene sequence possess a bootstrap value of 80% (Figure 1), presenting <5% GC (mol %) (45.2 ± 2.26%) [25,33], which could constitute a new clade named Swingsii. Phylogenetic analysis of 16S rRNA gene sequence revealed that *P. sanguinicancri* is closely related to *P. swingsii* [33]. However, this inclusion of these two species in a clade in the MLSA approaches has been accomplished at bootstrap values <75% (Figure 2, Supplementary Figures S3 and S4) and this not confirmed the proposed of this clade.

Only the inclusion of *P. aplysiae* is uncertain and constitutes the unique orphan clade. Several authors, on the basis of 16S rRNA gene sequence, revealed that *P. aplysiae* was closely related to *P. alginatilyticum* [35,37] and to *P. swingsii* [25], although these results are not based on MLSA analyses, and, therefore, it is necessary to include more strains in further phylogenetic studies to elucidate the inclusion of *P. aplysiae* in any clade.

### 3.3. Intra- and Interspecies Nucleotide Sequences Variation

The mean intra- and interspecies nucleotide sequence similarities for the different genes tested are shown in Table 2. The intraspecies gene similarities in the Damselae clade are high with six genes tested (>83%), regarding the geographical location and isolation host. For the other clades established in the present study, the intraspecies gene similarities were variable, with values between 44.0% and 99.9% for clade Phosphoreum; between 79.4% and 98.4% for clade Profundum; between 12.0% and 97.8% for clade Ganghwense; and between 15.8% and 99.2% for clade Leiognathi. 

The interspecies gene similarities are also shown in Table 2. Clade Damselae shares high mean similarities with clade Phosphoreum for 16S rRNA (97.0%) and *gapA* (87.8%) genes. High mean similarity percentage between clades Damselae and Profundum was obtained for the genes: 16S rRNA gene (96.3%), *gapA* (86.1%), *ftsZ* (82.3%) and *mreB* (84.9%). Clade Damselae shares a high mean similarity with clade Ganghwense only for 16S rRNA gene (96.3%). Finally, the new described clade Leiognathi shares a high mean similarity with clade Damselae for 16S rRNA gene (96.9%) and the genes *gapA* (87.4%) and *mreB* (84.4%). 

Clade Phosphoreum shares high mean similarities with clade Profundum for 16S rRNA gene (97.6%) and for the *gapA* and *gyrB* genes (91.0% and 77.7%, respectively). With the clade Ganghwense high mean similarities was only recorded for 16S rRNA gene (97.1%), whilst with clade Leiognathi, clade Phosphoreum shares high mean similarities for the genes 16S rRNA gene (98.3%), *gapA* (90.3%) and *gyrB* (80.2%). 

Clade Profundum shares high mean similarities with clade Ganghwense was only found for 16S rRNA gene (96.2%). In the case of the clade Leiognathi, clade Profundum shows high mean similarities for the genes 16S rRNA (97.1%), *gapA* (90.9%) and *mreB* (86.5%). Finally, clades Ganghwense and Leiognathi shares high mean similarities only for the gene 16S rRNA (97.3%). 

## 4. Conclusions

The MLSA has showed to be a robust technique suitable for elucidating phylogenetic relationships among *P. damselae* strains and between *P. damselae* and other species of the *Photobacterium* genus and hence, its use is necessary for taxonomy of this microbial group. By using this assay 22 from 31 of the described until now species of *Photobacterium* have been adequately discriminated and 5 clades have been proposed on the basis of MLSA approach. Two additional clades, Rosenbergii and Swingsii, were formed using the 16S rRNA gene as phylogenetic approach, although they are not confirmed by any MLSA methods. Thus, only *P*. *aplysiae* is not included in any cluster and it constitutes an orphan clade. All the new recently described species (validated or not) have also been clustered in the defined and proposed clades.

## Figures and Tables

**Table 1:** List of *Photobacterium* species including the habitats and geographic sources of isolation.

Species	Habitats	Geographic Sources
*P. aestuarii*	Tidal flat sediment	Yeongam Bay (R. Korea)
*P. alginatilyticum*	Bottom seawater	East China Sea
*P. angustum*	Seawater	North Pacific Ocean (20°30′ N 157°30′ E)
*P. aphoticum*	Seawater	Malvarrosa beach, Valencia (Spain)
*P. aplysiae*	Eggs of sea hare (*Aplysia kurodai*)	Mogiyeo (R. Korea)
*P. aquae*	Malabar grouper (*Epinephelus malabaricus*) in mariculture system	Tianjin (China)
*P. aquimaris*	Seawater	Sagami Bay (Japan)
*P. carnosum*	Packaged poultry meat	Germany
*P. damselae*	Damselfish (*Chromis punctipinnis*) skin ulcer ^a^, white perch (*Roccus americanus*)	California, Chesapeake Bay (USA)
*P. frigidiphilum*	Deep-sea sediments (1450 m)	Edison Seamount (western Pacific Ocean)
*P. gaetbulicola*	Tidal flat	Gung harbour (R. Korea)
*P. galatheae*	Mussel	Solomon Sea (Solomon Islands)
*P. ganghwense*	Seawater	Ganghwa Island (R. Korea)
*P. halotolerans*	Water from a subterranean saline lake	Lake Martel, Mallorca (Spain)
*P. iliopiscarium*	Intestines of fish (herring, coal fish, cod and salmon) living in cold seawater	Norway
*P. indicum*	Marine mud (400 m depth)	Indian Ocean
*P. jeanii*	Healthy corals (*Palythoa caribaeorum*, *Phyllogorgia dilatata* and *Merulina ampliata*)	Brazil and Australia
*P. kishitanii*	Light organs and skin of several marine fish species	Japan, Cape Verde, Hawaii, Florida, South Africa
*P. leiognathi*	Light organ of teleostean fish (*Leiognathus*)	Gulf of Thailand (Thailand)
*P. lipolyticum*	Intertidal sediment	Yellow Sea (R. Korea)
*P. lutimaris*	Tidal flat sediment	Saemankum (R. Korea)
*P. panuliri*	Eggs of spiny lobster (*Panulirus penicillatus*)	Andaman Sea (India)
*P. phosphoreum*	Skin of marine animals, intestines of marine fish, luminous organs, seawater	Hawaii (USA), Japan and other locations
*P. piscicola*	Skin and intestine of marine fish, spoiled packed cod	North Sea (The Netherlands), Denmark, Aberdeen Bay (UK)
*P. profundum*	Deep-sea sediment (5110 m)	Ryukyu Trench (24°15.23′ N 126°47.30′ E)
*P. proteolyticum*	Ocean sediment	Laizhou Bay (China)
*P. rosenbergii*	Tissue and water extracts of coral species	Magnetic Island (Australia)
*P. sanctipauli*	Coral (*Madracis decactis*)	St. Peter & St. Paul Archipelago (Brazil)
*P. sanguinicancri*	Crab (*Maja brachydactyla*) haemolymph, mussels (*Mytilus edulis*)	Spain, Netherlands
*P. swingsii*	Pacific oysters (*Crassostrea gigas*), crab (*Maja brachydactyla*) haemolymph	Mexico, Spain
*P. toruni*	Diseased redbanded seabream (*Pagrus auriga*)	Spain

^a^ Additional strains have been isolated from human puncture wound, diseased shark, diseased turtle, diseased fish, aquarium seawater and fish surface.

**Table 2:** Mean sequence similarities (%) between *Photobacterium* clades. D: Clade Damselae. (18 strains), Ph: Clade Phosphoreum (8 strains), P: Clade Profundum (5 strains), G: Clade Ganghwense (6 strains) and L: Clade Leiognathi (3 strains).

Genes	D	Ph	P	G	L
*16S rRNA*					
D	99.6				
Ph	97.0	99.9			
P	96.3	97.6	98.4		
G	96.3	97.1	96.2	97.8	
L	96.9	98.3	97.1	97.3	99.2
*gyrB*					
D	83.2				
Ph	70.7	91.7			
P	68.5	77.7	81.0		
G	65.9	73.8	72.7	79.1	
L	69.3	80.2	70.6	73.6	87.5
*gapA*					
D	91.4				
Ph	87.8	98.1			
P	86.1	91.0	88.4		
G	NA	NA	NA	75.0	
L	87.4	90.3	90.9	NA	96.9
*topA*					
D	84.5				
Ph	72.0	89.1			
P	69.9	60.9	79.4		
G	NA	NA	NA	70.5	
L	40.2	45.9	46.7	NA	15.8
*ftsZ*					
D	99.4				
Ph	52.6	44.0			
P	82.3	52.9	80.1		
G	2.5	14.0	4.9	15.2	
L	58.1	44.0	58.5	12.0	38.8
*mreB*					
D	98.2				
Ph	47.0	NA			
P	84.9	43.2	83.3		
G	25.1	20.5	22.9	NA	
L	84.4	43.1	86.5	22.1	90.5

NA: Not available.

## Supplementary Materials

The following are available online at http://www.mdpi.com/2076-2607/6/1/24/s1, Figure S1: Neighbor-joining phylogenetic tree based on 16S rRNA gene sequences, Figure S2: Maximum likelihood phylogenetic tree based on 16S rRNA gene sequences, Figure S3: Neighbor-joining phylogenetic tree based on based on 5 concatenated genes (*gyrB*, *gapA*, *topA*, *ftsZ*, and *mreB*), Figure S4: Maximum likelihood phylogenetic tree based on based on 5 concatenated genes (*gyrB*, *gapA*, *topA*, *ftsZ*, and *mreB*), Table S1: Primers used for the amplification of the housekeeping genes, Table S2: GenBank accession numbers of gene sequences used in this study, Table S3: Intraspecific phenotypic variation among the *Photobacterium damselae* strains.

Supplementary File 1
