# Supplementary material for: Description of New and Amended Clades of the Genus Photobacterium
Source: Microorganisms. 2018 Mar 12;6(1):24. doi: 10.3390/microorganisms6010024 (PMC5874638; doi:10.3390/microorganisms6010024)
Supplement: Supplementary File 1 [file microorganisms-06-00024-s001.zip › microorganisms-267692-supplementary/Supplementary Table S1rev.pdf]

Supplementary Table S1. Primers used for the amplification of the housekeeping genes.

Gene Target	Primer ID	Sequence 5'-3'	Reference
16S rRNA	63f	CAGGCCTAACACATGCAAGTC	[1]
	1387r	GGGCGGWGTGTACAAGGC	
gyrB	VgyrB325F	TACAAAGTATCGGGTGGTCT	[2]
	VgyrB1486R	ACGTCTGCGTCGGTCATGAT	
gapA	VgapA150F	AACTCACGGTCGTTTCAAC	[3]
	VgapA899R	CGTTGTCGTACCAAGATAC	
topA	VtopA400F	GAGATCATCGGTGGTGATG	[3]
	VtopA1200R	GAAGGACGAATCGCTTCGTG	
ftsZ	VftsZ75F	GTCGTTGAACACATGGTACG	[3]
	VftsZ800R	GCACCAGCAAGATCGATATC	
mreB	VmreB12F	ACTTCGTGGCATGTTTTC	[3]
	VmreB999R	CCGTGCATATCGATCATTTC	

References

1. Jung, S.Y.; Jung, Y.T.; Oh, T.K.; Yoon, J.H. *Photobacterium lutimaris* sp. nov., isolated from a tidal flat sediment in Korea. *Int. J. Syst. Evol. Microbiol.* **2007**, *57*, 332–336.
2. Thompson, F.L.; Thompson, C.C.; Naser, S.; Hoste, B.; Vandemeulebroecke, K.; Munn, C.; Bourne, D.; Swings, J. *Photobacterium rosenbergii* sp. nov. and *Enterovibrio coralii* sp. nov., vibrios associated with coral bleaching. *Int. J. Syst. Evol. Microbiol.* **2005**, *55*, 913–917.
3. Sawabe, T.; Kita-Tsukamoto, K.; Thompson, F.L. Inferring the evolutionary history of vibrios by means of multilocus sequence analysis. *J. Bacteriol.* **2007**, *189*, 7932–7936.
