# Supplementary material for: Description of New and Amended Clades of the Genus Photobacterium
Source: Microorganisms. 2018 Mar 12;6(1):24. doi: 10.3390/microorganisms6010024 (PMC5874638; doi:10.3390/microorganisms6010024)
Supplement: Supplementary File 1 [file microorganisms-06-00024-s001.zip › microorganisms-267692-supplementary/Supplementary Table S2.pdf]

Supplementary Table S2. GenBank accession numbers of gene sequences used in this study.

Photobacterium species	*gyrB*	*gapA*	*topA*	*ftsZ*	*mreB*
P. angustum ATCC 25915^T	AF136383	EF415490	DQ907457	UB33_RS14340	UB33_14050
P. aphoticum JCM 19237	JCM19237_6778	JCM19237_1433	JCM19237_4151	JCM19237_2573	JCM19237_2573
P. aplysiae CAIM 14	KU681312	KF666661	KF666751	KF666643	KF666707
P. aquae AE6^T	KF170932	ABT56_RS07980	ABT56_RS02285	ABT56_RS09240	ABT56_08970
P. aquimaris LC2-065^T	AB428879	AB428885	AB453690	AB453686	AB453694
P. carnosum TMW 2.2021^T	MF622950	NZ_NPIB01000012	NPIB01000001	NZ_NPIB01000008	NZ_NPIB01000008
P. damselae subsp. *damselae* ATCC 33539^T	AB298188	DQ907258	DQ907458	DQ907319	DQ907386
P. d. subsp. *damselae* CIP102761^T	VDA_RS13080	VDA_RS00465	VDA_RS07690	VDA_RS00045	VDA_003504
P. d. subsp. *damselae* C11080601R	**KU534149**[*]	**KU534142**	**KU534135**	**KU534128**	**KU534121**
P. d. subsp. *damselae* CECT 5064	**KU680722**	**KU680713**	**KU680703**	**KU680683**	**KU680693**
P. d. subsp. *damselae* D20040408U	**KU680714**	**KU680704**	**KU680694**	**KU680674**	**KU680684**
P. d. subsp. *damselae* H01100402O	**KU680715**	**KU680705**	**KU680695**	**KU680675**	**KU680685**
P. d. subsp. *damselae* H01100403D1	**KU534150**	**KU534143**	**KU534136**	**KU534129**	**KU534122**
P. d. subsp. *damselae* H09060401H	**KU534148**	**KU534141**	**KU534134**	**KU534127**	**KU534120**
P. d. subsp. *damselae* H14060501B	**KU680717**	**KU680707**	**KU680697**	**KU680677**	**KU680687**
P. d. subsp. *damselae* H14060501H	**KU680718**	**KU680708**	**KU680698**	**KU680678**	**KU680688**
P. d. subsp. *damselae* H14060501R	**KU680719**	**KU680709**	**KU680699**	**KU680679**	**KU680689**
P. d. subsp. *damselae* H22060601R	**KU534146**	**KU534139**	**KU534132**	**KU534125**	**KU534118**
P. d. subsp. *damselae* H29040401H	**KU680716**	**KU680706**	**KU680696**	**KU680676**	**KU680686**
P. d. subsp. *damselae* H29060602R	**KU534147**	**KU534140**	**KU534133**	**KU534126**	**KU534119**
P. d. subsp. *damselae* Lb07070501R	**KU534151**	**KU534144**	**KU534137**	**KU534130**	**KU534123**
P. d. subsp. *damselae* S04070503C	**KU534152**	**KU534145**	**KU534138**	**KU534131**	**KU534124**
P. d. subsp. *piscicida* ATCC 17911	**KU680721**	**KU680712**	**KU680702**	**KU680682**	**KU680692**
P. d. subsp. *piscicida* L09110601O	**KU680720**	**KU680710**	**KU680700**	**KU680680**	**KU680690**
P. frigidiphilum JCM 12947^T	AB298189	DQ907259	DQ907459	DQ907320	DQ907387
P. gaetbulicola Gung47^T	H744_RS10325	H744_RS12670	H744_RS22455	H744_RS12275	CP005974
P. galatheae S2753^T	EA58_08470	EA58_04580	EA58_11710	EA58_05035	EA58_05325
P. ganghwense DSM 22954	ABT57_22530	ABT57_18865	ABT57_01030	ABT57_19625	ABT57_19910
P. halotolerans DSM 18316^T	FN796485	H511_RS0118115	H511_RS0116995	H511_RS0118555	H511_RS0118845
P. iliopiscarium ATCC 51760^T	AY455878	EF415498	UB38_RS01660	UB38_RS13985	UB38_12625

Photobacterium species	*gyrB*	*gapA*	*topA*	*ftsZ*	*mreB*
P. indicum NBRC 14233[T]	AB159514	EF415489	DQ907462	DQ907323	DQ907390
P. jeanii R-40508[T]	LVHF01000030	KU702662	GU065214	GU065217	GU065220
P. kishitanii ATCC BAA-1194[T]	UB40_RS16325	EF415500	AB453692	AB453688	AB453696
P. leiognathi subsp. *leiognathi* ATCC 25521[T]	AY455879	EF415492	DQ907463	UB42_RS05010	UB42_05295
P. leiognathi subsp. *mandapamensis* ATCC 27561[T]	AY455883	EF415494	AB453693	AB453689	AB453697
P. lipolyticum DSM 16190[T]	AB298194	DQ907264	DQ907464	DQ907325	DQ907392
P. lutimaris CAIM 1851	KF666688	KF666663	KF666753	KF666644	KF666709
P. marinum AK15[T]	C942_RS18665	C942_RS12610	C942_RS02280	C942_RS07915	C942_04764
P. phosphoreum ATCC 11040[T]	AY455875	EF415496	UB41_RS00885	UB41_RS05575	UB41_12100
P. piscicola NCCB 100098[T]	KF206123	KF938492	KX943609	KX943610	KX943611
P. profundum 3TCK	CH724137	CH724136	CH724136	CH724136	CH724136
P. proteolyticum YIC13-12[T]	BIT28_08625	BIT28_RS16330	BIT28_22380	BIT28_16855	BIT28_17150
P. rosenbergii LMG 22223[T]	AB298197	DQ907267	DQ907467	DQ907328	DQ907395
P. sanctipauli A-394[T]	CQ09_RS26825	CQ09_RS11505	CQ09_RS17135	CQ09_RS23280	CQ09_RS05785
P. sanguinicancri CAIM 1827[T]	KU681315	KU681323	KU681319	KU169268	KU681322
P. swingsii CAIM 1393[T]	AB733_RS18275	KF666664	KF666754	KF666645	KF666710
P. toruni CECT 9189[T]	KX855662	KX855663	KX855664	KX855665	KX855666
Salinivibrio costicola ATCC 33508[T]	D481_RS19680	D481_RS0112370	D481_RS20830	D481_RS0101800	D481_RS0101345

[*]Bold text corresponds to gene sequences that were sequenced in this study.
