# Supplementary material for: Description of New and Amended Clades of the Genus Photobacterium
Source: Microorganisms. 2018 Mar 12;6(1):24. doi: 10.3390/microorganisms6010024 (PMC5874638; doi:10.3390/microorganisms6010024)
Supplement: Supplementary File 1 [file microorganisms-06-00024-s001.zip › microorganisms-267692-supplementary/Supplementary Table S3rev.pdf]

Supplementary Table S3. Intraspecific phenotypic variation among the *Photobacterium damselae* strains.

**Test**	***P. damselae* subsp. *damselae* (n=15)**	***P. damselae* subsp. *piscicida* (n=3)**
**Lysine decarboxylase**	d	-
**Motility**	+	-
**Acetoine production**	d	d
**Nitrate production**	+	-
**Amylase**	d	d
**Gelatinase**	d	-
**Lipase**	d	+
**Haemolysin production**	d	-
**Acids from:**		
**Sucrose**	-	w
**Melibiose**	+	-
**L-arabinose**	-	d
**D-cellobiose**	+	-
**D-galactose**	+	w
**Maltose**	+	-
**D-trehalose**	+	-
**Utilization of:**		
**Glycogen**	+	-
**Tween-40**	+	-
**N-acetyl-D-galactosamine**	+	-
**ß-methyl-D-glucoside**	+	-
**D-raffinose**	+	-
**D-sorbitol**	+	-
**Succinic acid**	+	-
**D-L- lactic acid**	+	-
**Bromosuccinic acid**	+	-
**Succinamic acid**	+	-
**L-alanyl glycine**	+	-
**L-asparagine**	+	-
**L-aspartic acid**	+	-
**L-glutamic acid**	+	-
**L-serine**	+	-
**Glycerol**	+	-
**α-D-glucose 1-phosphate**	+	-

d: variable result among strains

w: weak positive
