# Supplementary figures and images for: Description of New and Amended Clades of the Genus Photobacterium
Source: Microorganisms. 2018 Mar 12;6(1):24. doi: 10.3390/microorganisms6010024 (PMC5874638; doi:10.3390/microorganisms6010024)

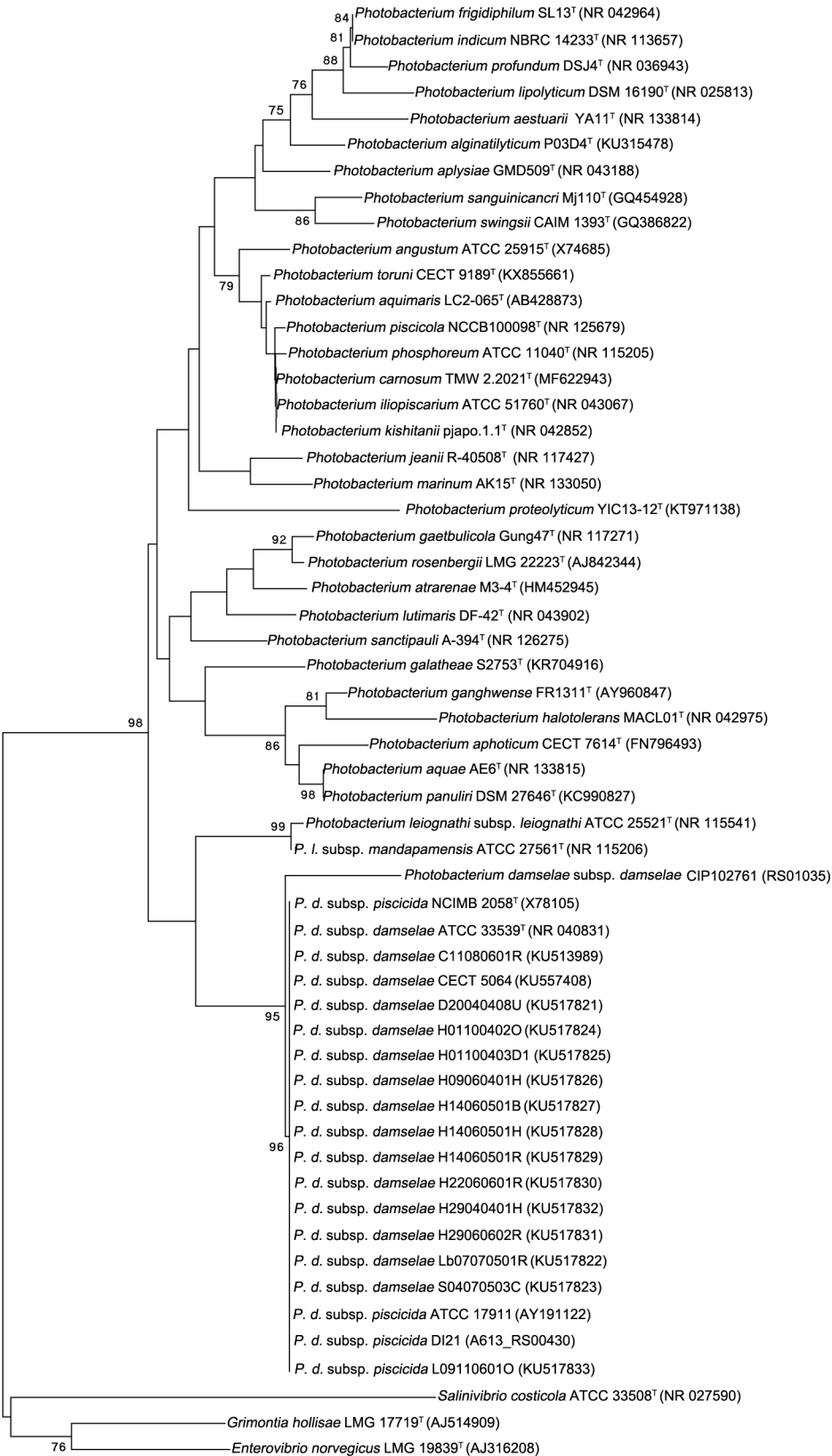

0.01

Supplement: Supplementary File 1 [file microorganisms-06-00024-s001.zip › microorganisms-267692-supplementary/SFigureS1.pdf]

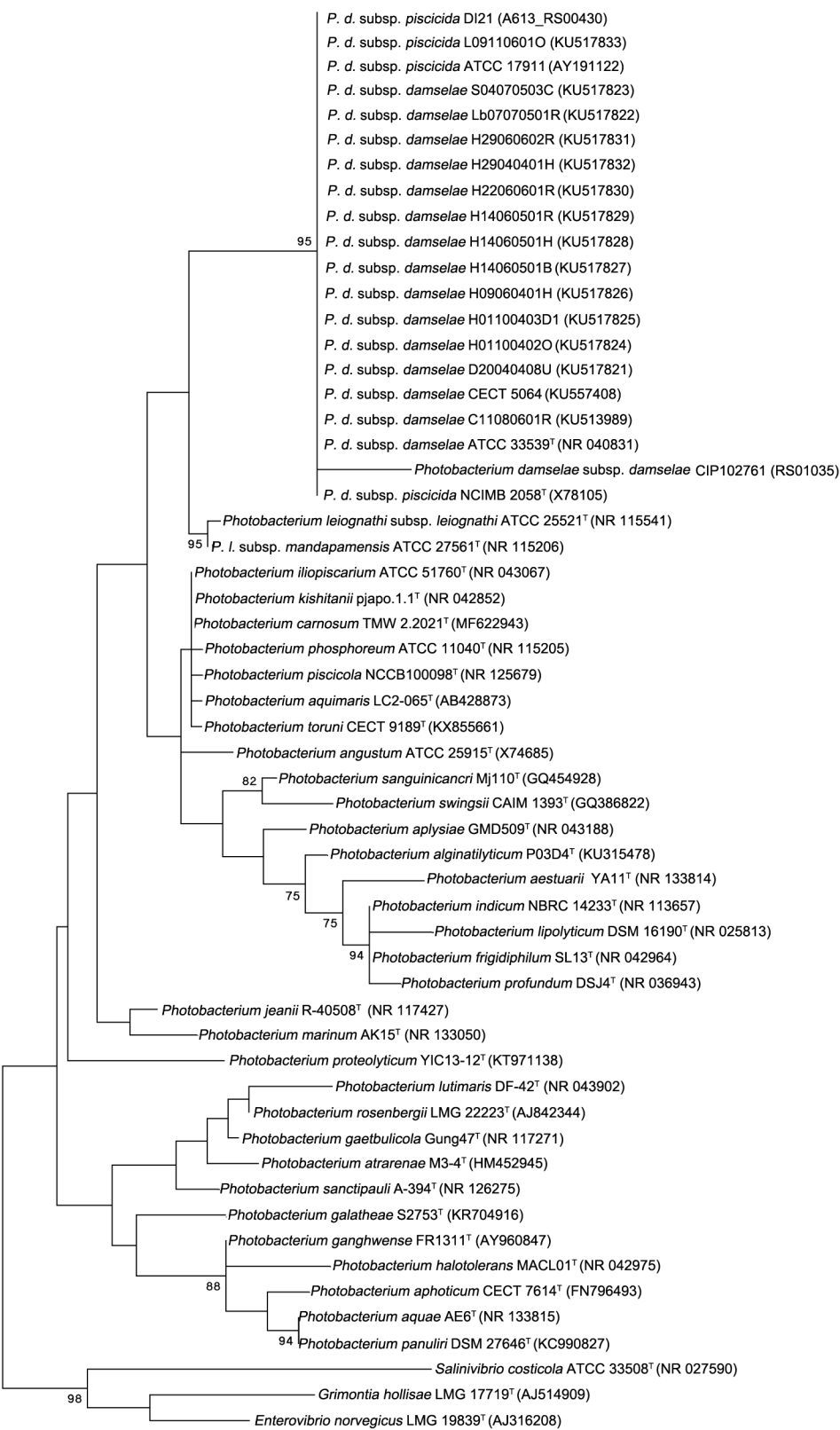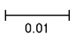

Supplement: Supplementary File 1 [file microorganisms-06-00024-s001.zip › microorganisms-267692-supplementary/SFigureS2.pdf]

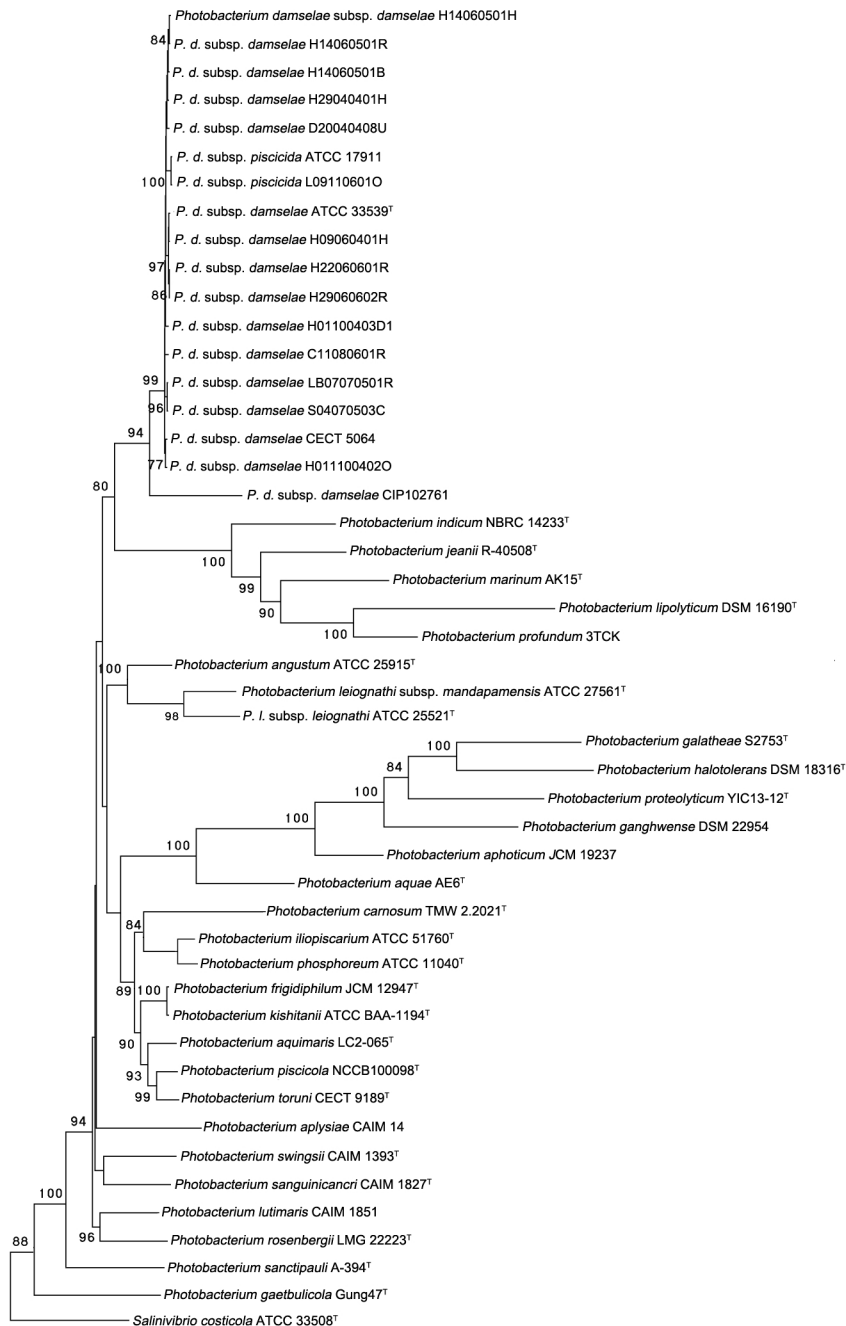

0.05

Supplement: Supplementary File 1 [file microorganisms-06-00024-s001.zip › microorganisms-267692-supplementary/SFigureS3.pdf]

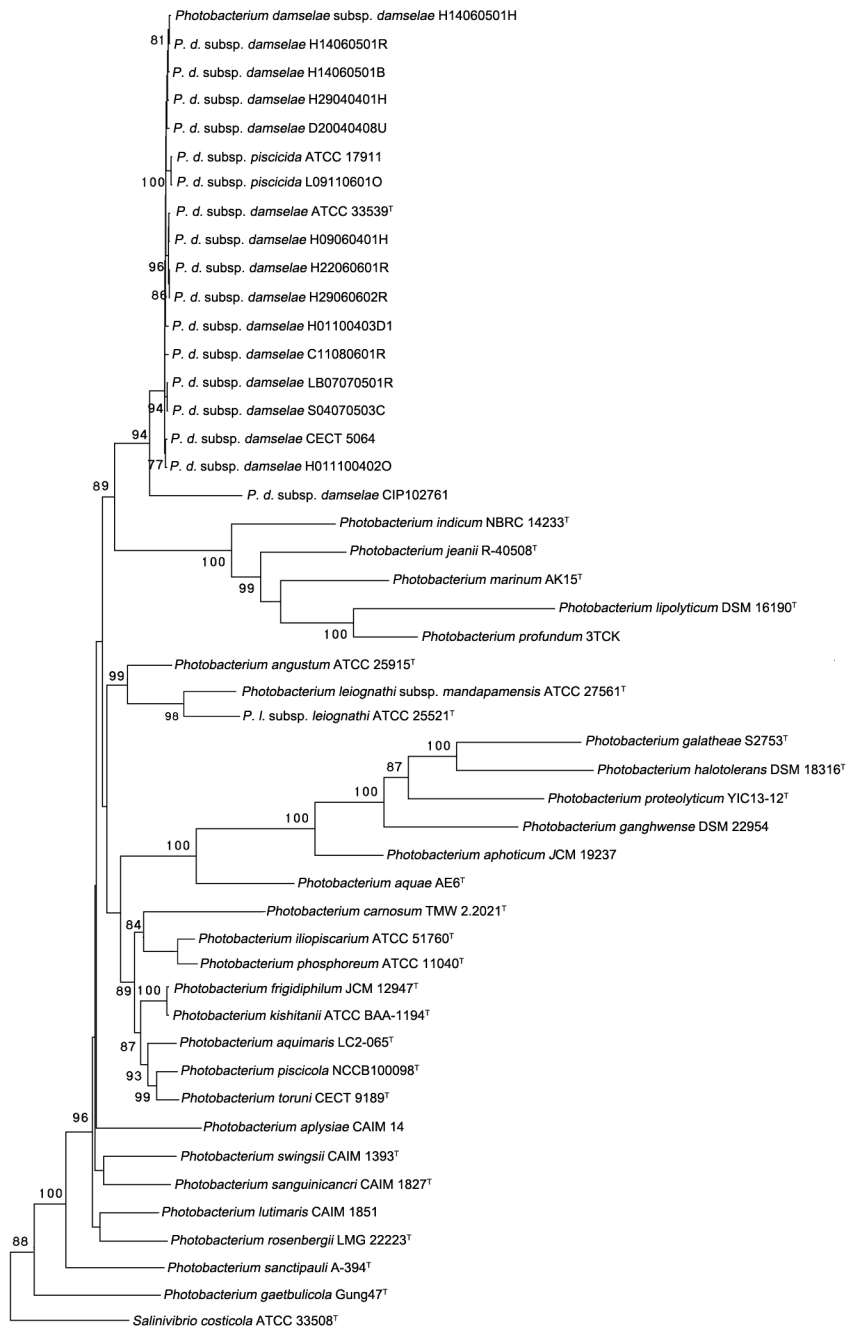

0.05

Supplement: Supplementary File 1 [file microorganisms-06-00024-s001.zip › microorganisms-267692-supplementary/SFigureS4.pdf]
